# Hydroquinone 5-*O*-Cinnamoyl Ester of Renieramycin M Suppresses Lung Cancer Stem Cells by Targeting Akt and Destabilizes c-Myc

**DOI:** 10.3390/ph14111112

**Published:** 2021-10-30

**Authors:** Nattamon Hongwiangchan, Nicharat Sriratanasak, Duangdao Wichadakul, Nithikoon Aksorn, Supakarn Chamni, Pithi Chanvorachote

**Affiliations:** 1 Cell-Based Drug and Health Products Development Research Unit, Faculty of Pharmaceutical Sciences, Chulalongkorn University, Bangkok 10330, Thailand; nattamon.chula@gmail.com (N.H.); nicharat.sri@outlook.com (N.S.); 2 Pharmaceutical Sciences and Technology Graduate Program, Faculty of Pharmaceutical Sciences, Chulalongkorn University, Bangkok 10330, Thailand; 3 Department of Pharmacology and Physiology, Faculty of Pharmaceutical Sciences, Chulalongkorn Univesity, Bangkok 10330, Thailand; 4 Department of Computer Engineering, Faculty of Engineering, Chulalongkorn University, Bangkok 10330, Thailand; duangdao.w@chula.ac.th; 5 Department of Clinical Pathology, Faculty of Medicine Vajira Hospital, Navamindradhiraj University, Bangkok 10300, Thailand; nithikoon@nmu.ac.th; 6 Department of Pharmacognosy and Pharmaceutical Botany, Chulalongkorn University, Bangkok 10330, Thailand; supakarn.c@pharm.chula.ac.th; 7 Natural Products and Nanoparticles Research Unit (NP2), Faculty of Pharmaceutical Sciences, Chulalongkorn University, Bangkok 10330, Thailand

**Keywords:** lung cancer, cancer stem cell, Akt, mTOR, c-Myc, renieramycin M, Oct4, Nanog, Sox2

## Abstract

Cancer stem cells (CSCs) are distinct cancer populations with tumorigenic and self-renewal abilities. CSCs are drivers of cancer initiation, progression, therapeutic failure, and disease recurrence. Thereby, novel compounds targeting CSCs offer a promising way to control cancer. In this study, the hydroquinone 5-*O*-cinnamoyl ester of renieramycin M (CIN-RM) was demonstrated to suppress lung cancer CSCs. CIN-RM was toxic to lung cancer cells with a half-maximal inhibitory concentration of around 15 µM. CIN-RM suppressed CSCs by inhibiting colony and tumor spheroid formation. In addition, the CSC population was isolated and treated and the CSCs were dispatched in response to CIN-RM within 24 h. CIN-RM was shown to abolish cellular c-Myc, a central survival and stem cell regulatory protein, with the depletion of CSC markers and stem cell transcription factors ALDH1A1, Oct4, Nanog, and Sox2. For up-stream regulation, we found that CIN-RM significantly inhibited Akt and consequently decreased the pluripotent transcription factors. CIN-RM also inhibited mTOR, while slightly decreasing p-GSK3β (Ser9) but rarely affected the protein kinase C (PKC) signal. Inhibiting Akt/mTOR induced ubiquitination of c-Myc and promoted degradation. The mechanism of how Akt regulates the stability of c-Myc was validated with the Akt inhibitor wortmannin. The computational analysis further confirmed the strong interaction between CIN-RM and the Akt protein with a binding affinity of −10.9 kcal/mol at its critical active site. Taken together, we utilized molecular experiments, the CSC phenotype, and molecular docking methods to reveal the novel suppressing the activity of this compound on CSCs to benefit CSC-targeted therapy for lung cancer treatment.

## 1. Introduction

Cancer stem cells (CSCs) are a leading cause of cancer aggressiveness that enhances the ability of cancer to disseminate. They are the small section that carries asymmetric division to remain the constant proportion and self-renewal properties. This unique population has been identified as a tumor initiator that progresses cancer [1,2]. Recent studies have highlighted the CSC population as a critical regulator of disease relapse, as CSCs have a very high detoxification ability and augmented drug resistance pathways; therefore, targeting CSCs is recognized as a promising way to control cancer [3].

CSCs exhibit biological activities and stem cell properties through several pluripotent transcription factors, such as Oct4, Nanog, and Sox2 [4]. Among the prominent regulators of pluripotency, the protein kinase B (Akt)/c-Myc axis has garnered increasing interest. Akt plays an essential role in various aspects of tumor growth, survival, and therapeutic resistance in numerous types of cancer [5,6]. c-Myc is a proto-oncogene transcription factor that regulates several downstream signaling pathways. The functions of c-Myc include cell cycle progression, survival metabolism, and stem cell activity [7,8]. Overexpression of the c-Myc protein results in aggressiveness and therapeutic resistance of cancers because of over-activated stemness properties [8,9]. The cellular availability of c-Myc is dependent on the stability of the protein which is controlled by Akt. Akt promotes the stability of c-Myc by inhibiting phosphorylation at threonine 58 (Thr58), which prevents protein degradation [10,11]. In addition, mTOR regulates c-Myc-driven tumorigenesis [12] and controls c-Myc stability [13].

A series of studies have demonstrated the cooperation between c-Myc and other self-renewal transcription factors, including Nanog, Sox2, and Oct4. These three self-renewal transcription factors possess predominant stemness activity [14,15]. c-Myc cooperates with other factors to regulate self-renewal. However, recent studies have demonstrated that c-Myc plays a role as a regulator of other self-renewal transcription factors by inducing the transcription and activity of the proteins [14]. Hence, inhibiting Akt which is upstream of various CSC-related regulators, is a promising strategy.

Marine-derived compounds are interesting biologically active compounds in cancer therapy. These compounds have complex structures that interact with various biomolecular targets to suppress or promote biological functions for treatment purposes [16]. Natural marine compounds and their synthetic derivatives have been investigated in clinical trials [17]. Marine-derived anticancer drugs, such as trabectedin and cytarabine, have been approved by the United States Food and Drug Administration [18]. According to recent studies, the tetrahydroisoquinoline family and marine/microbial alkaloids such as renieramycins, ecteinascidins, saframycins, safracins, and naphthyridinomycins exhibit anticancer properties against cancer cells [19].

Renieramycin M is a marine alkaloid in the bistetrahydroisoquinolinequinone family that has been isolated from the Thai blue sponge, *Xestospongia* sp. Renieramycin M exhibits anticancer activities in several cancer cells, such as lung, breast, and colon [20]. Interestingly, this compound not only suppresses non-stem cancer cells but also suppresses CSCs in lung carcinoma [21]. Hydroquinone 5-*O*-cinnamoyl ester of renieramycin M (CIN-RM), which contains an additional cinnamoyl ester on C-5 and a hydroxyl moiety at the C-8 of ring A, displays a better apoptosis-inducing potency in H292 lung cancer cells [22]. CIN-RM was synthesized by a two-step chemical modification of renieramycin M involving palladium catalyzed hydrogenation and Steglich esterification [23]. However, no study has reported on the suppression of CSCs by CIN-RM. This research aims to investigate the potential effect of CIN-RM on the CSCs of lung cancer and elucidate the underlying mechanism, which involves Akt/mTOR regulating the c-Myc pathway.

## 2. Results

### 2.1. Semi-Synthesis of Hydroquinone 5-O-Cinnamoyl Ester of Renieramycin M (CIN-RM)

**Figure 1:**
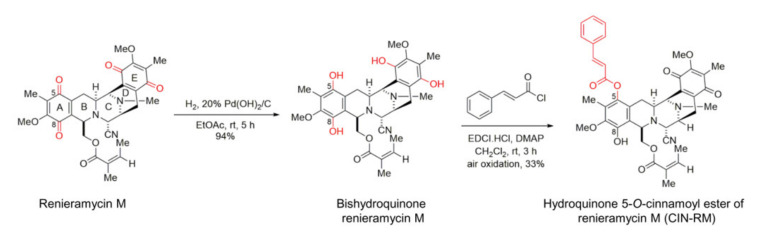
Semi-synthesis of hydroquinone 5-*O*-cinnamoyl ester of renieramycin M (CIN-RM).

CIN-RM was prepared from remieramycin M based on the previously reported two-step chemical modification involving palladium catalyzed hydrogenation and esterification (Figure 1) [23]. Under this procedure, the quinone moieties on ring A and E of renieramycin M were reduced to the hydroxyl groups to become bistetrahydroquinone renieramycin M. Esterification occurred at the C-5 hydroxyl group only based upon the sterical hindrance to furnish hydroquinone monoester renieramycin M. Subsequent oxidation by air provided CIN-RM in an expectable yield (33%).

### 2.2. Selective Cytotoxicity of CIN-RM in Human Lung Cancer Cells

**Figure 2:**
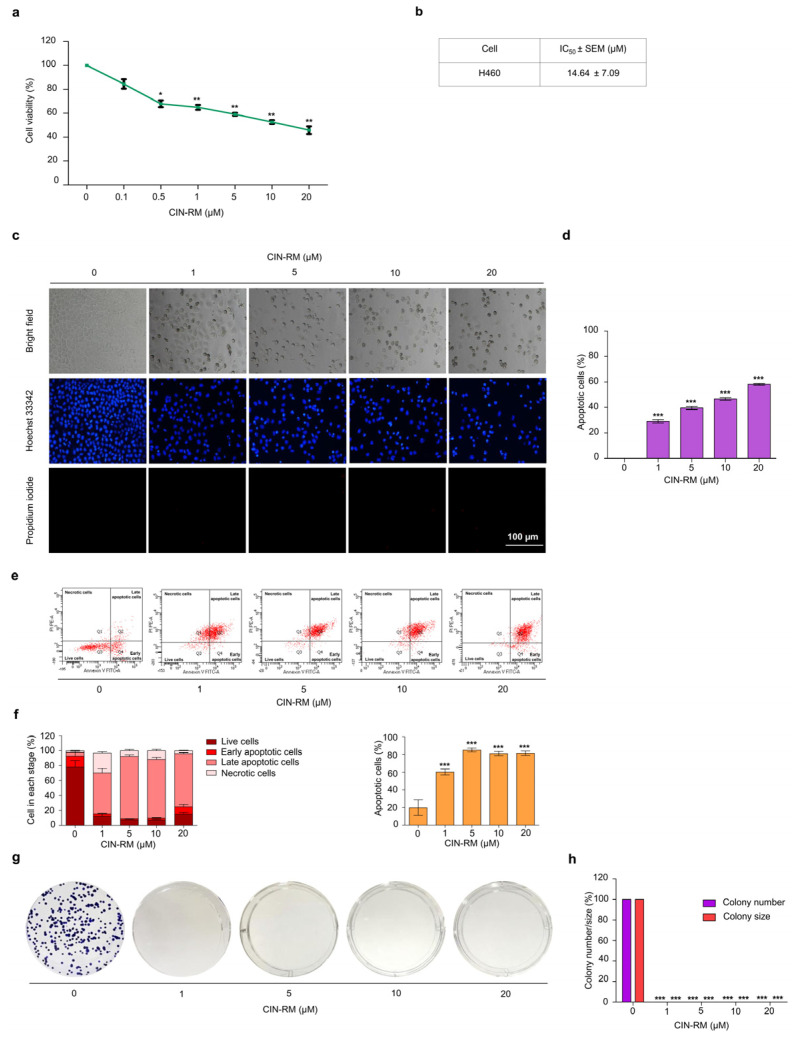
The anticancer activity of CIN-RM was indicated by (**a**) the significant reduction of % cell viability in human lung cancer H460 cells with CIN-RM at 0–20 μM for 24 h. (**b**) The half maximal inhibitory concentration (IC_50_) of CIN-RM in H460 cells was 14.64 ± 7.09 μM. (**c**) Apoptotic H460 cells were detected by Hoechst 33342/PI staining and visualized by fluorescence microscopy. (**d**) The percentage of apoptotic cells in CIN-RM-treated cells was analyzed. (**e**,**f**) Cells were treated with various concentrations of CIN-RM (0–20 μM) for 24 h, and apoptosis was evaluated by annexin V-FITC/PI staining. (**g**,**h**) Cells were treated with various concentrations of CIN-RN (0–20 µM) for 24 h before being subjected to forming colonies for 7 days, then the colonies were stained with crystal violet. All data are presented as the mean ± SEM (*n* = 3). * *p* < 0.05, ** *p* < 0.01, and *** *p* < 0.001 compared with untreated cells.

To elucidate the anticancer potential of CIN-RM (Figure 1), we determined the cytotoxic profile of CIN-RM in lung cancer H460 cells. The cells were incubated with CIN-RM (0–20 µM) for 24 h. The results showed that CIN-RM significantly reduced the viability of H460 cells (Figure 2a) with a half maximal inhibitory concentration (IC_50_) value of 14.64 ± 7.09 μM (Figure 2b). Further investigations of CSC-targeting activity of the compound were performed in H460 cells treated with 0–20 μM CIN-RM. Apoptosis is characterized by condensation and fragmentation of DNA. Therefore, we examined whether the majority of the cytotoxic effects caused by CIN-RM were related to apoptosis. Hoechst 33342 staining was used to evaluate the nuclear morphology of the CIN-RM-treated cells. H460 cells were treated with 0–20 µM CIN-RM for 24 h. In addition, propidium iodide (PI) fluorescent dye was used to detect necrosis; however, no PI-positive cells were detected at the tested CIN-RM concentrations. These results reveal that 1–20 µM CIN-RM induced apoptosis cell death as indicated by the clear presence of DNA condensation and/or fragmentation (Figure 2c,d). The reduction in cell viability detected by MTT correlated well with the induction of apoptosis at the same concentrations. The apoptosis inducing activity of CIN-RM was confirmed by annexin-V-FITC/PI staining with flow cytometry analysis. CIN-RM significantly induced apoptotic cell death at 1–20 µM (Figure 2e,f). Our results suggest that apoptosis was the main mode of cell death of the CIN-RM treated cells.

It is widely accepted that CSCs can escape apoptosis in response to chemotherapy. Next, we tested whether CIN-RM had this effect on a resistant cell population in the modified colony formation assay. Surviving H460 cells after a 24 h CIN-RM (1, 5, 10, and 20 μM) treatment were subjected to a clonogenic assay without further treatment. Crystal violet-stained colonies representing the capability to reproduce a new cancer colony from a single cell were shown in Figure 2g,h. It was shown that the resistant cells receiving CIN-RM at 1–20 µM could not form the colonies (Figure 2g,h).

### 2.3. CIN-RM Attenuates Anchorage-Independent Growth and Suppresses CSC Spheroid Formation

**Figure 3:**
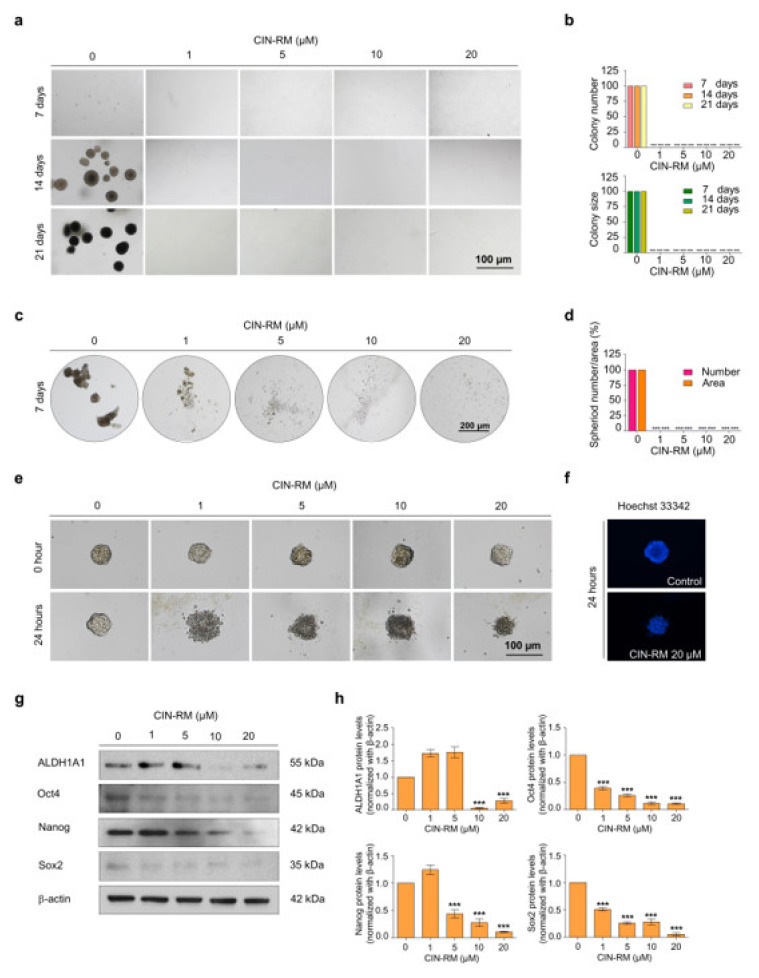
CIN-RM suppresses anchorage-independent growth and cancer stem cell (CSC)-like phenotype of human lung cancer cells. (**a**,**b**) Cells were pretreated with CIN-RM for 24 h, and the surviving cells were subjected to an anchorage-independent growth assay. (**c**,**d**) Cells were pretreated with CIN-RM for 24 h and allowed to form primary spheroids for 7 days, and the spheroid of the CSC population was determined. (**e**,**f**) The CSC single spheroid was treated with toxic concentrations of CIN-RM, and the apoptosis cell death was analyzed with Hoechst 33342. (**g**,**h**) H460 cells were treated with various concentrations (0–20 µM) of CIN-RM for 24 h. The expression of ALDH1A1, Oct4, Nanog, and Sox2 were determined by Western blotting. β-actin was determined to confirm equal loading of the samples. Densitometry of each protein was calculated and the results were presented as relative protein levels when compared with untreated control. Data are represented as the mean ± SEM (*n* = 3). *** *p* < 0.001 compared with untreated cells.

It was previously reported that the process of anchorage-independent growth of cancer cells reflects the anoikis-resistant capability of malignant tumor cells [24]. To test whether CIN-RM could suppress such cancer cell survival and the ability to grow under detached conditions, H460 cells were treated with CIN-RM for 24 h. The surviving cells were collected and grown for 7, 14, and 21 days in soft agar for the anchorage-independent growth assay (Figure 3a). The number and size of the growing cancer colonies were determined and calculated relative to those of the untreated control. The results indicated that the CIN-RM-pretreated cells exhibited decreased anchorage-independent growth compared with the untreated control (Figure 3b). As illustrated in Figure 3b, the numbers of H460 cell colonies decreased significantly in response to the 1, 5, 10, and 20 µM CIN-RM treatment, and the percentage of the colony size in response to 1, 5, 10, and 20 µM CIN-RM was 100%. These results suggest that the CIN-RM treatment may affect the signaling pathways influencing the growth of cancer cells in the detached condition.

As the ability of cancer cells to form tumor spheroids has been used to reflect the CSC phenotype, we studied the effect of CIN-RM on spheroid formation. H460 cells were treated with various concentrations of CIN-RM (0–20 µM) for 24 h, and the cells were subjected to the spheroid formation assay. The primary spheroids were captured under a microscope after 7 days (Figure 3c). The results show that the untreated control cells had a high ability to form primary tumor spheroids, whereas the cells treated with CIN-RM exhibited a complete abolition of tumor spheroids at any dose (Figure 3c,d). To further confirm this CSC suppressing activity, a CSC-rich population was established from a secondary spheroid of control cells. The CSC spheroids were seeded in ultralow attachment 96-well plates at a density of one spheroid per well. The spheroids were treated with CIN-RM (0–20 µM) for 24 h. The results showed that the untreated control spheroid survived and maintained the integrity of the tumor spheroid, whereas the CIN-RM-treated cells revealed a dissociated pattern of spheroids (Figure 3e). Hoechst 33342 staining further revealed the apoptosis character of DNA fragmentation and/or DNA condensation in the CIN-RM treated cells (Figure 3f).

After showing that CIN-RM suppressed CSC properties, we next confirmed this result by evaluating the CSC markers in CIN-RM-treated cells. CSCs highly express self-renewal transcription factors and detoxifying enzymes, such as ALDH1A1, Oct4, Nanog, and Sox2 [4]. The H460 cells were treated with various concentrations of CIN-RM (0*–*20 µM) for 24 h and expression of the ALDH1A1, Oct4, Nanog, and Sox2 proteins was measured. The results showed that Oct4 and Sox2 were dramatically decreased at 1 µM of CIN-RM, while Nanog decreased significantly at 5 µM. ALDH1A1 decreased significantly at 10 µM (Figure 3g,h) when compared with the untreated control.

### 2.4. CIN-RM Suppression of CSC Is Mediated via Akt Inhibition

**Figure 4:**
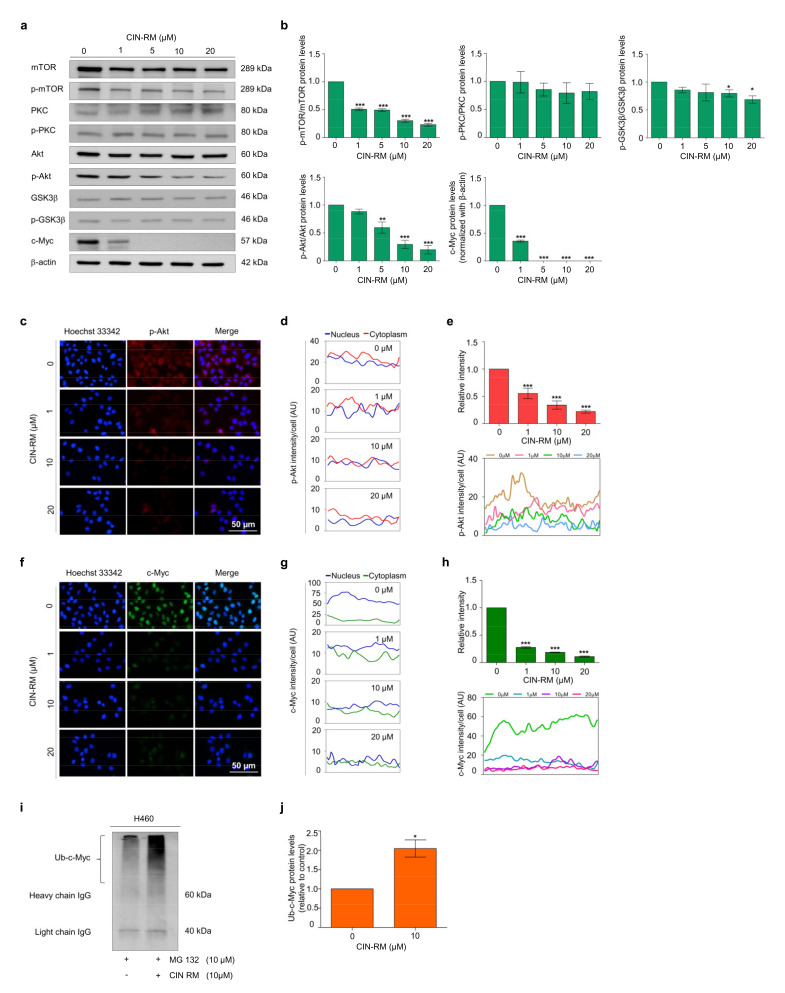
Effect of CIN-RM on Akt/c-Myc signaling pathway. (**a**) H460 cells were treated with various concentrations (0–20 µM) of CIN-RM for 24 h and the expression levels of mTOR, p-mTOR, PKC, p-PKC, GSK3β, p-GSK3β, Akt, p-Akt, and c-Myc protein were investigated by Western blotting. (**b**) Blots were reprobed with β-actin to confirm equal loading of samples. The immunoblot signals were quantified by densitometry. (**c**,**f**) H460 cells were treated with CIN-RN at toxic concentrations for 12 h. The cellular levels of p-Akt and c-Myc were determined by immunofluorescence analysis. (**d**,**g**) The fluorescence intensity of the nucleus and cytoplasm were analyzed by ImageJ software. (**e**,**h**) The fluorescence intensity was analyzed by ImageJ software. (**i**) H460 cells were treated with 10 µM MG132 for 1 h followed by 10 µM CIN-RM for 3 h. The specific c-Myc protein was immunoprecipitated using an antibody against c-Myc. The immunocomplex was evaluated by immunoblotting using ubiquitin antibodies. (**j**) Densitometry of Ub-c-Myc protein complex level was calculated. Values are presented as means ± SEM (*n* = 3). * *p* < 0.05, ** *p* < 0.01, and *** *p* < 0.001 compared with untreated cells.

Akt and c-Myc play major roles in cell survival, proliferation, and stem cell properties. It is known that Akt controls the degradation of c-Myc by ubiquitin proteasomal degradation [11]. To investigate whether CIN-RM suppresses CSCs through the Akt/c-Myc signaling pathway, H460 cells were treated with various concentrations (0–20 μM) of CIN-RM and investigated by Western blot analysis (Figure 4a). The results indicated that the expression of p-Akt, p-mTOR, and c-Myc decreased significantly, while the expression of p-GSK3β (Ser9) decreased slightly compared to those of non-treated control cells (Figure 4b), suggesting that the CSC-suppressive activity of the compound may, at least in part, act via Akt/c-Myc inhibition. Moreover, p-PKC and PKC levels were evaluated to exclude Akt/PKC signaling. The results demonstrated that the expression of p-PKC did not decrease significantly in response to CIN-RM (Figure 4a,b).

We further confirmed the inhibitory effect of CIN-RM on the Akt/c-Myc signaling pathway using an immunofluorescence staining assay. H460 cells were treated with 0–20 μM of CIN-RM for 12 h before being incubated with p-Akt and c-Myc primary antibodies. Overall, p-Akt and c-Myc fluorescence intensity decreased significantly in the cytoplasm and nucleus (Figure 4c–h). Interestingly, while the level of p-Akt was evenly distributed in both cell compartments (Figure 4d), c-Myc was predominantly located in the nucleus of untreated control cells (Figure 4g).

Emerging research has shown that Akt affects the degradation of c-Myc via the ubiquitin proteasomal pathway [25]. Enhanced c-Myc degradation has also been linked to a reduction of CSC transcription factors, including Sox2, Oct4, and Nanog [26]. This study further investigated whether downregulation of Akt by CIN-RM resulted in degradation of the c-Myc ubiquitin proteasomal pathway. H460 cells were pretreated with 10 µM of the proteasomal inhibitor MG132 for 1 h followed by 10 μM of CIN-RM for 3 h. The c-Myc-ubiquitin complex was evaluated by an immunoprecipitation assay. Figure 4i–j indicate that the level of the c-Myc-ubiquitin complex increased approximately two-fold in CIN-RM-treated cells. These results demonstrate that suppressing CSCs with CIN-RM occurred through Akt-dependent c-Myc destabilization.

**Figure 5:**
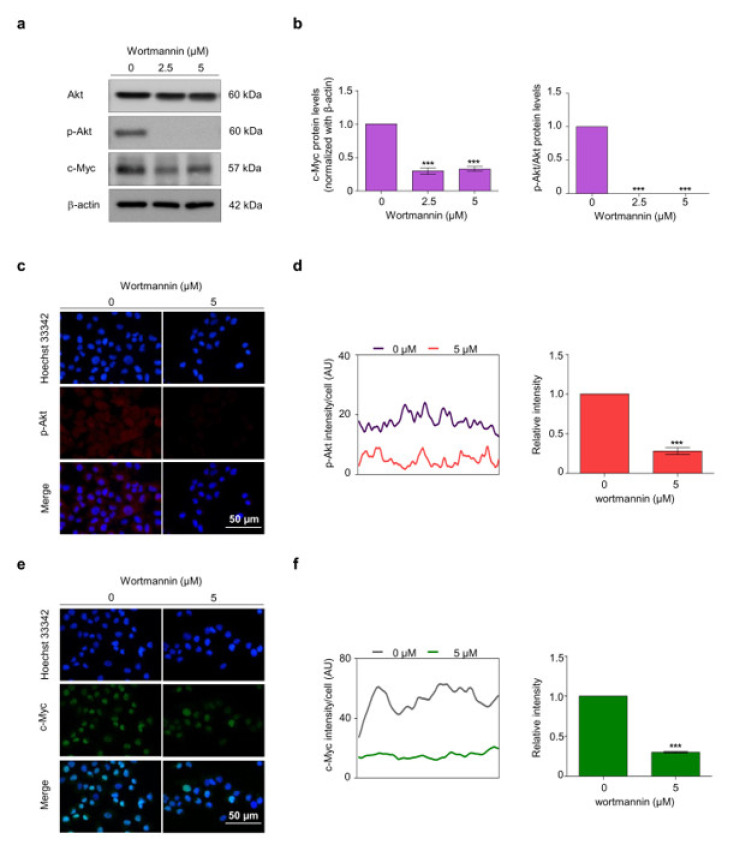
To demonstrate the relationship between the Akt signaling pathway and c-Myc, wortmannin, an Akt inhibitor, was used. (**a**) H460 cells were treated with 2.5 and 5 μM of wortmannin for 12 h and the expression levels of Akt, p-Akt, and c-Myc were analyzed by Western blotting. (**b**) Levels of Akt, p-Akt, and c-Myc were analyzed by densitometric analysis. (**c**,**e**) H460 cells were treated with 5 μM of wortmannin for 12 h. The treated cells were probed with anti-p-Akt and c-Myc. Hoechst33342 was used to identify nuclear localization. The expression of p-Akt and c-Myc were investigated using immunofluorescence. (**d**,**f**) The fluorescence intensity was analyzed using ImageJ software. Values are presented as means ± SEM (*n* = 3). *** *p* < 0.001 compared with untreated cells.

The PI3K inhibitor wortmannin was used to validate the regulation in H460 cells to bolster the finding that Akt regulates the stability of c-Myc. Cells were treated with wortmannin at 2.5 and 5 μM for 12 h and the Akt, p-Akt, and c-Myc protein levels were determined. Wortmannin significantly decreased the p-Akt and c-Myc levels, while the Akt protein levels remained unchanged (Figure 5a,b). The immunofluorescence staining assay confirmed suppression of Akt by the PI3K inhibitor and further revealed that c-Myc was consequently suppressed (Figure 5c–f).

### 2.5. CIN-RN Suppression of CSC on Other Lung Cancer Cells

**Figure 6:**
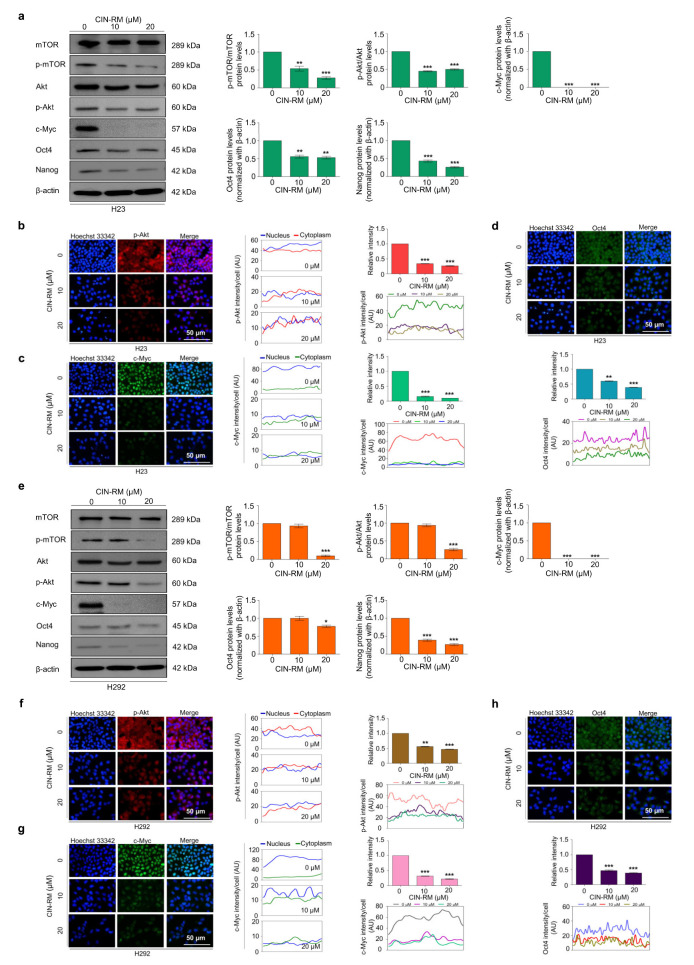
Effect of CIN-RM on lung cancer H23 (**a**–**d**) and H292 (**e**–**h**) cells. (**a**) H23 and (**e**) H292 cells were treated with various concentrations (0, 10, and 20 µM) of CIN-RM for 24 h and the expression levels of mTOR, p-mTOR, Akt, p-Akt, c-Myc, Oct4, and Nanog proteins were investigated by Western blotting. Blots were reprobed with β-actin to confirm equal loading of samples. The immunoblot signals were quantified by densitometry. (**b**,**c**,**d**) H23 cells were treated with CIN-RN at toxic concentrations (0, 10, and 20 µM) for 12 h. The cellular levels of p-Akt, c-Myc, and Oct4 were determined by immunofluorescence analysis. The fluorescence intensity of the nucleus and cytoplasm were analyzed by ImageJ software. (**f**,**g**,**h**) H292 cells were treated with CIN-RN at toxic concentrations (0, 10, and 20 µM) for 12 h. The cellular levels of p-Akt, c-Myc, and Oct4 were determined by immunofluorescence analysis. The fluorescence intensity of the nucleus and cytoplasm were analyzed by ImageJ software. Values are presented as means ± SEM (*n* = 3). * *p* < 0.05, ** *p* < 0.01, and *** *p* < 0.001 compared with untreated cells.

To confirm the effect of CIN-RM suppressing CSCs through the Akt/c-Myc signaling pathway on other lung cancer cells, H23 and H292 cells were treated with various concentrations of CIN-RM (0, 10, and 20 μM) and investigated for key regulatory proteins by Western blot analysis. The results indicated that the expression of p-Akt, p-mTOR, c-Myc, Oct4, and Nanog significantly decreased in response to CIN-RM compared with untreated control cells in both cells (Figure 6a,e), indicating that the CSC-suppressive activity of the CIN-RM on Akt/c-Myc is not cell type-specific. We further confirmed the inhibitory effect of CIN-RM on the Akt/c-Myc pathway using an immunofluorescence staining assay. H23 and H292 cells were treated with 0, 10, and 20 μM of CIN-RM for 12 h. Overall, p-Akt, c-Myc, and Oct4 fluorescence intensity significantly decreased in the CIN-RM treated cells (Figure 6b–d,f–h).

### 2.6. Molecular Docking Simulation Demonstrates the Interaction of CIN-RM with the Akt Protein

**Figure 7:**
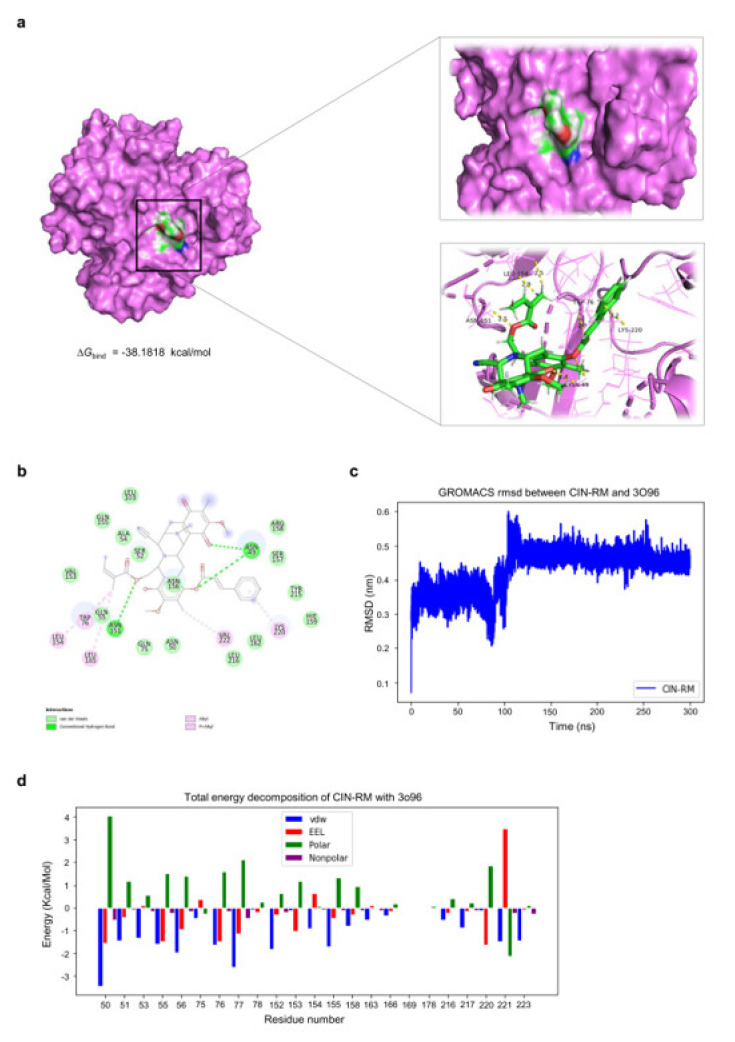
Docked model depicting the interaction of CIN-RM with the Akt protein. (**a**) Binding mode and docking energy of CIN-RM bound to the binding site of Akt taken from the MD study. (**b**) 2-dimensional (2D) of the interaction detail between CIN-RM and Akt. (**c**) The RMSD plot for the interaction of the CIN-RM/Akt complex during 300 ns of molecular dynamic simulation. (**d**) Total energy decomposition of the CIN-RM/Akt complex using the gmx_MMPBSA program.

To evaluate the feasibility of direct interaction between CIN-RM and Akt, we performed a molecular docking simulation of CIN-RM with Akt (PDB ID: 3O96), with a resolution of 2.70 Å. The binding affinity obtained from AutoDock Vina was −10.9 kcal/mol. As illustrated in Figure 7a,b, CIN-RM potentially binds with Akt through several interactions, whereby the key potential interaction includes conventional hydrogen bonding (residues ASN49 and ASN151) and Pi-Alkyl interactions (residues LEU154, TRP76, LEU165, VAL222, and LYS220). The root mean square deviation (RMSD) was established to assess the binding stability of the complex. The binding stability of this complex based on RMSD values was found to be of high stability (Figure 7c). The binding free energy MM/GBSA (ΔG_bind_) score and its energy components of the CIN-RM/Akt complex are represented in Table 1. According to the results, ΔG_bind_ of the CIN-RM/Akt complex was calculated to be −38.1818 kcal/mol. From the polar structure of CIN-RM (Figure 7d), the molecular mechanics energy (∆E_MM_) showed that van der Waals interaction (∆E_vdW_) was the major force of the CIN–RM/Akt complex (∆E_vdW_ = −63.3923 ± 2.7583 kcal/mol).

## 3. Discussion

CSCs are a unique sub-population within tumors that are linked to low-rated successful treatments. CSCs have several cellular defensive mechanisms to escape conventional treatments [27]. The remaining CSCs that are not eliminated by chemotherapy initiate and promote cancer relapse [28]. Therefore, the molecules targeting CSCs as well as the mechanism maintaining cancer stemness should offer a novel promising treatment for cancer [29]. CSCs commonly overexpress specific CSC markers, such as ALDH1A1, and possess a high level of pluripotent transcription factors, including Nanog, Sox2, and Oct4. The CSCs in many cancers are enhanced by Akt and its downstream regulator, c-Myc [4,5]. As the stem cell-rich population of cancers has a highly active Akt signaling mechanism and elevated c-Myc [5,30], these two proteins have been recognized as important drug targets for cancer treatment [31]. In this study, we demonstrated the activity of CIN-RM in suppressing lung cancer CSCs with a possible underlying compound action mechanism.

Marine-derived compounds from various sources have been demonstrated to inhibit CSCs. For example, 5-*O*-acetyl-renieramycin T induces apoptosis and decreases expression of the CSC markers (CD44 and CD133), and decreases the Nanog stem cell transcription factor via the Akt signaling pathway [32]. An extract of the marine sponge *Crambe crambe* (CR) strongly reduces pancreatic and prostate CSCs [33]. RM suppressed CSC-like phenotypes in H460 lung cancer cells [21]. CIN-RM (Figure 1) was semi-synthesized from renieramycin M which is isolated from the Thai blue sponge *Xestospongia* sp. [23]. A previous study demonstrated that CIN-RM shows potential as an anticancer agent by triggering apoptosis-inducing factors (AIF) and a caspase cascade leading to apoptotic cell death in lung cancer [22]. Our study revealed that the CIN-RM treatment resulted in a significant induction of apoptotic cell death and inhibited cell proliferation (Figure 2c–h). We have added up the novel information that CIN-RM significantly obstructed anchorage-independent cell growth and inhibited the ability to form tumor spheroids (Figure 3a–d) and eradicated the formed spheres (Figure 3e,f).

ALDH1A1, Nanog, Oct4, and Sox2 are reported stem cell markers in lung cancer [4,34]. Overexpression of ALDH represents highly tumorigenic, cancer cell cloning properties [35] and reveals chemo-resistance [36]. Moreover, Nanog, Sox2, and Oct4 are pluripotent transcription factors regulating self-renewal capacity. In our study, we discovered that CSCs suppressed the activity of CIN-RM by inhibiting ALDH1A1 and the pluripotency transcription factors (Figure 3g,h). For the up-stream regulatory mechanism, CSC transcription factors were shown to be activated via several pathways including Akt. It was previously shown that Akt directly regulates Oct4 and Sox2 activity [37,38,39,40]. Akt increases the stability of the Oct4 protein by phosphorylating Oct4 at threonine 235. Phosphorylated Oct4 enters the nucleus and interacts with Sox2, which in turn activates the transcription of Nanog [41].

In addition, c-Myc, a major downstream target of Akt, accompanies Oct4, Nanog, and Sox2 to promote self-renewal in CSCs [14]. c-Myc is a co-factor of Oct4/Sox2/KLF4 during pluripotent stem cell reprogramming [42]. Akt regulated the stability of c-Myc via a GSK3β-dependent mechanism [10]. Similarly, the stability of c-Myc is controlled by the Akt/mTOR pathway [13]. mTOR inhibits Ser62 dephosphorylation on c-Myc by hindering PP2A activity [43] leading to the stabilization of c-Myc. The expression levels of the p-Akt, p-mTOR, and c-Myc proteins significantly decreased in response to CIN-RM (Figure 4a,b). The results of the immunofluorescence assay demonstrated in the same manner that CIN-RM significantly decreased the intensity of p-Akt and c-Myc in the cytoplasm and nucleus, respectively, of H460 cells (Figure 4d,g). The stability of c-Myc is dependent on the control by Akt in protecting c-Myc proteasomal degradation by inhibiting GSK3β [11]. Therefore, disrupting the Akt signal could result in indirect suppression of CSCs by destabilizing c-Myc. From these results, CIN-RM only slightly affected p-GSK3β (Ser9), as well as p-PKC, but strongly decreased p-mTOR expression (Figure 4a,b), suggesting that CIN-RM regulated c-Myc degradation through the Akt/mTOR pathway.

Inhibiting Akt is a therapeutic target for various cancers, including lung cancer [38]. Several critical binding sites affect Akt activity. Akt inhibitors are allosteric inhibitors responsible for binding to the PH domain or kinase domain leads. The PH domain is the target site of phosphatidylinositol (3,4,5)-trisphosphate (PIP3). PIP3 is generated from PI3K to recruit Akt to the plasma membrane [44]. The other critical site is the phosphorylation site. Akt is phosphorylated at the catalytic (kinase) domain (containing Thr 308) and the hydrophobic C-terminal tail (containing Ser 473), which is the active form of the protein [45]. In the current research, anticancer agents have been developed using Akt inhibitors focused on allosteric inhibition [46]. We further tested our hypothesis by performing molecular docking using Akt1 (as a target for the CIN-RM compound) with an allosteric inhibitor model (PDB code: 3O96). The results revealed that CIN-RM acted as an allosteric inhibitor of Akt. The binding affinity of CIN-RM with Akt was −10.9 kcal/mol and the binding free energy MM/GBSA (Δ*G*_bind_) was −38.1818 kcal/mol, demonstrating that CIN-RM has the potential to bind to the Akt protein. Interestingly, the CIN-RM structure (Supplementary Materials) was closely bound to Akt through hydrogen bonds at residues ASN49 and ASN151; there were many Pi-Alkyl interactions with the residues, including LEU154, TRP76, LEU165, VAL222, and LYS220 (Figure 7). Currently, there are several allosteric inhibitors of Akt have been reported, such as MK-2206, ALPs, OSU-A9, PH-316, PHT-427, and DM-PIT-1 [46].

CIN-RM exhibited a potential CSC-targeting activity by inhibiting Akt. As CSCs have been shown to drive cancer progression, drug resistance, metastasis, and relapse, this compound may offer novel approaches for the improvement of highly resistant and relapsing cancers. In addition, the Akt inhibitory effect of the compound may be useful for many human cancers in which Akt is over-activated with highly drug-resistant characteristics. Furthermore, this novel model could be developed as a new molecular cancer treatment targeting Akt.

## 4. Materials and Methods

### 4.1. Non-Small Cell Lung Cancer Cell Lines and Cultures

Non-small cell lung cancer cells used in the experiments were H460, H23, and H292 and were obtained from the American Type Culture Collection (Manassas, VA, USA). H460, H23, and H292 cells were grown in Roswell Park Memorial Institute (RPMI) 1640 medium containing 10% fetal bovine serum (FBS), 2 mM L-glutamine, and 100 units/mL each of penicillin and streptomycin under 5% carbon dioxide (CO_2_) at 37 °C conditions in an incubator.

### 4.2. Semi-Synthesis of Hydroquinone 5-O-Cinnamoyl Ester of Renieramycin M (CIN-RM)

Hydroquinone 5-*O*-cinnamoyl ester of renieramycin M (CIN-RM) was synthesized from renieramycin M that was isolated from the Thai blue sponge *Xestospongia* sp. The blue sponge was collected by scuba diving in the vicinity of Si-Chang Island at a depth of 3–5 m with permission from the Department of Fisheries, Ministry of Agriculture and Cooperatives, Thailand (0510.2/8234, Date 28th October 2019). The fresh *Xestospongia* sp. was kept frozen at −20 °C until use. The extraction, pretreatment with potassium cyanide, purification, and structural determination were performed exactly as previously described to obtain renieramycin M as the orange solid with an isolation yield of 0.01% of the dry sponge [47]. Next, renieramycin M (40.0 mg, 0.070 mmol) was dissolved in ethyl acetate (20 mL). The reaction was added to 20% palladium hydroxide on carbon, Pd(OH)_2_/C (20.0 mg, 50% *w*/*w*). A hydrogen balloon was attached to the reaction flask. The heterogeneous reaction was stirred vigorously at room temperature (25 °C) and 1 atm for 5 h. The reaction was filtered through a pad of celite and washed with ethyl acetate (10 mL, 3 times) and chloroform (10 mL, 3 times), respectively. The filtrates were combined and concentrated under reduced pressure to yield bistetrahydroquinone renieramycin M as a colorless solid. The resulting bistetrahydroquinone renieramycin M (38.2 mg, 0.066 mmol) was dissolved in dry dichloromethane (15 mL). The colorless reaction mixture was added to 1-ethyl-3-(3-dimethylaminopropyl) carbodiimide hydrochloride (EDCI.HCl, 22.6 mg, 0.118 mmol) and N, N-4-dimethylaminopyridine (DMAP, 14.4 mg, 0.118 mmol) to afford a yellow-brown solution. Then, cinnamoyl chloride (19.6 mg, 0.118 mmol) was added. The resulting yellow reaction mixture was stirred under an argon atmosphere at room temperature for 3 h. After completion, the reaction was quenched by the addition of water (10 mL) and immediately oxidized by air. The organic layer was separated by a separatory funnel and the aqueous layer was extracted with chloroform (10 mL, 3 times). The organic layers were combined, washed with a saturated sodium chloride solution (30 mL), dried over anhydrous sodium sulfate, filtered, and concentrated under reduced pressure. The obtaining crude product was purified by the silica gel flash column chromatography using a solution of hexane:ethyl acetate (3:1) as an eluent to afford the corresponding hydroquinone 5-*O*-cinnamoyl ester of renieramycin M (CIN-RM) as a brown amorphous solid with an isolation yield of 33% (16.2 mg). The structural determination was confirmed by spectroscopic techniques and spectroscopic data were matched with the previous report [23] (Data S1). The CIN-RM was kept in a desiccator at room temperature until use. For the biological assay, CIN-RM was prepared as a solution in dimethyl sulfoxide (DMSO) and diluted with Roswell Park Memorial Institute (RPMI) 1640 Medium cell culture in 10% FBS to obtain the desired concentration. The final concentration of DMSO was less than 0.5% of the solution, which showed no signs of cytotoxicity.

### 4.3. Reagents and Antibodies

Roswell Park Memorial Institute (RPMI) 1640 medium, penicillin/streptomycin, fetal bovine serum (FBS), phosphate-buffered saline (PBS), L-glutamine, and trypsin-EDTA were acquired from Gibco (Grand Island, NY, USA). Dimethyl sulfoxide (DMSO), 3-(4,5-dimethylthiazol-2-yl)-2,5-diphenyltetrazoliumbromide (MTT), propidium iodide (PI), Hoechst 33342, Triton X-100, bovine serum albumin (BSA), MG132, and paraformaldehyde were obtained from Sigma-Aldrich, Co. (St. Louis, MO, USA). Agarose was obtained from Bio-Rad Laboratories (Hercules, CA, USA). RIPA buffer and wortmannin were acquired from Cell Signaling Technology, Inc. (Danvers, MA, USA). The primary antibodies used in the experiments including β-Actin (#4970), Akt (#9272), phosphorylated Akt or p-Akt (#4060), c-Myc (#5605), PKC (#2056), p-PKC (#9375), GSK3β (#9323), p-GSK3β (#9832), mTOR (#2983), p-mTOR (#5536), Nanog (#4903), Oct4 (#2840), Sox2 (#3579), and ALDH1A1 (#36671) were acquired from Cell Signaling Technology (Danvers, MA, USA). The primary antibody ubiquitin (ab7780) was purchased from Abcam (Cambridge, UK). The respective secondary antibodies, anti-rabbit IgG (#7074), and anti-mouse (#7076) were also obtained from Cell Signaling Technology (Danvers, MA, USA).

### 4.4. Cytotoxicity Assay

To study the cytotoxicity of CIN-RM on NSCLC cell lines (H460), the MTT colorimetric assay was used. H460 1.5 × 10^4^ cells/well were cultured in a 96-well tissue culture plate with 100 µL/well RPMI in 10% FBS at 37 °C under 5% CO_2_ in an incubator overnight. Cells in 96-well plates were treated with various concentrations of CIN-RM (0, 0.1, 0.5, 1, 5, 10, 20 µM) for 24 h. After that, cells were incubated with 0.4 mg/mL MTT for 3 h at 37 °C. Then, 100% DMSO was added to dissolve the formazan crystals. The intensity of the MTT product was measured at 570 nm by a microplate reader (Anthros, Durham, NC, USA). The percentage of cell survival (% cell viability) and IC_50_ were calculated as described in the manufacturer’s protocol (7sea Biotech, Shanghai, China). Cell viability (%) = (ODexperiment−ODblank)/(ODcontrol−ODblank) × 100%.

### 4.5. Apoptosis Assay

Apoptotic and necrotic cells were investigated using the Annexin V-FITC apoptosis kit (Thermo Fisher Scientific, Waltham, MA, USA). The treated cells were trypsinized and suspended in 70 µL of 1X binding buffer. Then, the cells were incubated with Annexin V/FITC in the dark at room temperature for 15 min. Then, the binding buffer was added up to 400 µL and the cells were stained with PI before performing a flow cytometry assay. The cells were analyzed with BD FACSDiva 8.0.2 flow cytometry systems.

### 4.6. Nuclear Staining Assay

Apoptosis and necrosis cell deaths were analyzed with a Hoechst 33342 and PI fluorescent DNA co-staining assay. The cells were seeded 1 × 10^4^ cells/well in 96 well plates and incubated overnight. Then, the cells were treated with CIN-RM at various concentrations (0–20 µM) for 24 h. After that, cells were incubated with 10 µg/mL Hoechst 33342 and 5 µg/mL PI for 30 min at 37°C. Then, cells were visualized by fluorescence microscopy (Nikon ECLIPSE Ts2) and the analysis was evaluated by ImageJ software.

### 4.7. Colony Formation Assay

H460 cells were pretreated with various concentrations (0–20 µM) of CIN-RM for 24 h. Next, CIN-RM-treated cells were detached and seeded at approximately 300 cells/well onto a 6-well plate and let them form colonies at 37 °C for 7 days. The cells were fixed with 4% paraformaldehyde for 30 min at room temperature, followed by staining with crystal violet solution at room temperature for 30 min and washed with tap water. The colony number and size were investigated by OpenCFU software.

### 4.8. Anchorage-Independent Growth Assay

The soft agar colony formation assay was used to determine anchorage-independent cell growth. The cells were pretreated with various concentrations (0–20 µM) of CIN-RM for 24 h. For the preparation of the agar, a 1:1 ratio mixture of RPMI medium containing 10% FBS and 1% agarose was added to a 24-well plate to form a bottom layer. An upper layer contained 8 × 10^3^ living cells/mL in the agarose gel with 10% FBS and 0.3% agarose. When the upper layer was solidified, RPMI medium containing 10% FBS was added and then incubated at 37°C. Phase-contrast images of colony formation were taken on days 7, 14, and 21 of treatment using a phase-contrast microscope (Olympus IX51 with DP70, Melville, NY, USA). The colony number and size were investigated by ImageJ software.

### 4.9. Spheroid Formation Assay

H460 cells were pretreated with various concentrations (0–20 µM) of CIN-RM for 24 h. The cells were detached and seeded approximately 2.5 × 10^3^ cells/well into a 6-well ultralow attachment plate with serum-free RPMI medium and incubated. Spheroid formation was determined after 7 days using a phase-contrast microscope (Nikon ECLIPSE Ts2). The analysis was evaluated by ImageJ software.

Meanwhile, H460 cells were seeded approximately 2.5 × 10^3^ cells/well into a 6-well ultralow attachment plate with serum-free medium and incubated for 7 days to form primary spheroids. Then, the primary spheroids were suspended into single cells and seeded onto a 96-well ultralow attachment plate with serum-free medium for 14 days to form secondary single spheroids. After that, the spheres were treated with various concentrations of CIN-RM (0–20 µM) and incubated for 24 h in an environment of 37 °C with 5% CO_2_. At 24 h after treatment, apoptosis cell death was analyzed with Hoechst 33342 and imaged using phase-contrast microscopy (Nikon ECLIPSE Ts2, Tokyo, Japan).

### 4.10. Immunofluorescence

H460, H23, and H292 cells were seeded in 96-well plates at a density of 8 × 10^3^ cells/well and incubated overnight. Then, H460 cells were treated with various concentrations (0–20 µM) of CIN-RM and 5 µM of wortmannin. H23 and H292 cells were treated with 0, 10, and 20 µM of CIN-RM and then incubated for 12 h. Next, cells were fixed with 4% paraformaldehyde for 15 min, followed by permeabilization by 0.5% of Triton X-100 in PBS for 5 min, and then blocking with 10% of FBS in 0.1% of Triton X-100 for 1 h at room temperature. Primary antibodies of c-Myc and p-Akt at a proportion of 1:200 in 10% FBS were applied before overnight incubation at 4 °C. After that, Alexa Fluor 488 IgG secondary antibody was added and incubated for 1 h in the dark at room temperature. Hoechst 33342 was used to stain cell nuclei, which were then visualized under a fluorescent microscope (Nikon ECLIPSE Ts2, Tokyo, Japan) and the analysis was evaluated by ImageJ software.

### 4.11. Immunoprecipitation Assay

H460 cell lines were pretreated with 10 μM of MG132 for 1 h followed by 10 μM of CIN-RM for 3 h. The treated cells were obtained and lysed with RIPA buffer. The magnetic beads from Dynabeads™ Protein G Immunoprecipitation Kit were washed with washing buffer and incubated with c-Myc primary antibody (Ab) in binding buffer for about 10 min. Then, the protein lysate was added to the bead-Ab complex suspension overnight at 4°C. Then, the complex was washed 3 times with 100 μL washing buffer. The supernatant was discarded and then the elution buffer was added for detaching the Ab-Ag complex from the beads. Finally, Western blot analysis was used to evaluate the ubiquitinated c-Myc protein.

### 4.12. Western Blot Analysis

Western blotting is a technique for detecting specific proteins in a sample. Cells were seeded at a density of 4 × 10^5^ cells/well in 6 well plates overnight. Cells were treated with CIN-RM at various concentrations (0–20 µM) for 24 h. Then, cells were washed with PBS (on ice) and incubated on ice for 30 min with 1X RIPPA 60 µL containing 10× RIPA buffer 100 µL, protease inhibitors (PI) 100 µL, PMSF 10 µL, and Triton X 10 µL. Protein content was analyzed using a BCA protein assay. The extracted proteins were separated with gel electrophoresis using 7.5–15% SDS-PAGE (Sodium dodecyl sulfate polyacrylamide gel). After that, the proteins were transferred from the gel to the polyvinylidene difluoride (PVDF) membrane, blocked with milk medium (Tris-HCl (pH 7.5) 25 mM, NaCl 125 mM, and 0.05% Tween20 (TBST) 0.05%) and 5% nonfat dry milk powder for 2 h, and incubated overnight with primary antibodies that were specific to the proteins (ALDH1A1, Nanog, Oct4, Sox2, Akt, p-Akt, c-Myc, PKC, p-PKC, GSK3β, p-GSK3β, mTOR, p-mTOR, and beta-actin). Then, the membranes were washed with TBST 3 times and then incubated with the secondary antibodies for 2 h at room temperature. Immunoreactive proteins were detected with the chemiluminescent evaluation system and subsequently exposed by Chemiluminescent ImageQuant LAS4000. Protein bands were analyzed using the ImageJ software.

### 4.13. Computational Akt Modelling and Molecular Docking

The binding of CIN-RM to AKT was investigated through molecular docking. Preparing the structure, the structure of CIN-RM was created and optimized using ChemBioDraw [48] and converted into pdb format using openbabel [49] to obtain the effective structure. The crystal structure of Human AKT1 with an Allosteric Inhibitor (PDB ID: 3O96; resolution at 2.70 Å) [50] was obtained from Protein Data Bank (PDB). We then used AutoDockTools to convert all pdb files to pdbqt files [51]. AutoDock vina was used to operate the docking calculation of the CIN-RM to the ATP binding pocket [52]. The absence of amino acid residues of 3O96 was completed by using the Swiss-PdbViewer program for the molecular dynamics (MD) simulations procedure [53]. We added the hydrogen atoms to the CIN-RM structure and created the mol2 files, topology files using Avogadro [54] and ACPYPE-AnteChamber [55] programs, respectively. In this experiment, the general AMBER force field (GAFF) [56] was used as the force field for ligand and AMBER ff14SB [57] was used as the force field for protein. We then used the TIP3P water model to solvate the system [58] and neutralized the system by adding Na+ and Cl- ions. V-rescale [59] was used in the temperature coupling method (the coupling constant: 0.1 ps). The Particle Mesh Ewald (PME) algorithm [60] was used as a standard method implemented for electrostatic interaction. Then, setting the short-range van der Waals (rvdw), neighbor list (rlist), and electrostatic (rcoulomb) cutoffs to 12 angstroms. All bond lengths were constrained by using the LINCS algorithm [61]. The time step was performed to 0.002 ps. The equilibration of the complex was performed in NVT and then NPT ensembles, (each time step: 100 ps) specifically, the molecular dynamics (MD) simulation was operated using GROMACS 2020.4 for 300 ns [62]. The stability between CIN-RM and the structure of 3O96 protein was measured by the root mean square deviation (RMSD) using gmx rms in GROMACS. We then calculated the binding free energy between receptor and ligand by the MM/GBSA method [63] in gmx_MMPBSA program [64] version 1.1.1. The PyMOL molecular graphics program (Schrödinger, Inc.) was used to visualize 3D molecular structure products for publication-quality images, and Discovery studio visualizer [65] was used to predict interaction and visualize 2D interactions.

### 4.14. Statistical Analysis

The results from three independent experiments (*n* = 3) were presented as means ± standard deviation for each group. Statistical differences between groups were analyzed using an analysis of variance (ANOVA), followed by individual comparisons with Schefft’s post-hoc test. For a two-group comparison, a t-test analysis was calculated. The statistic was calculated by SPSS software program version 16 (SPSS Inc., Chicago, IL, USA). The p-value of less than 0.05 was considered statistically significant. * *p* < 0.05, ** *p* < 0.01, and *** *p* < 0.001. GraphPad Prism 5 was used to create graphs in this experiment (GraphPad Software, San Diego, CA, USA).

## 5. Conclusions

**Figure 8:**
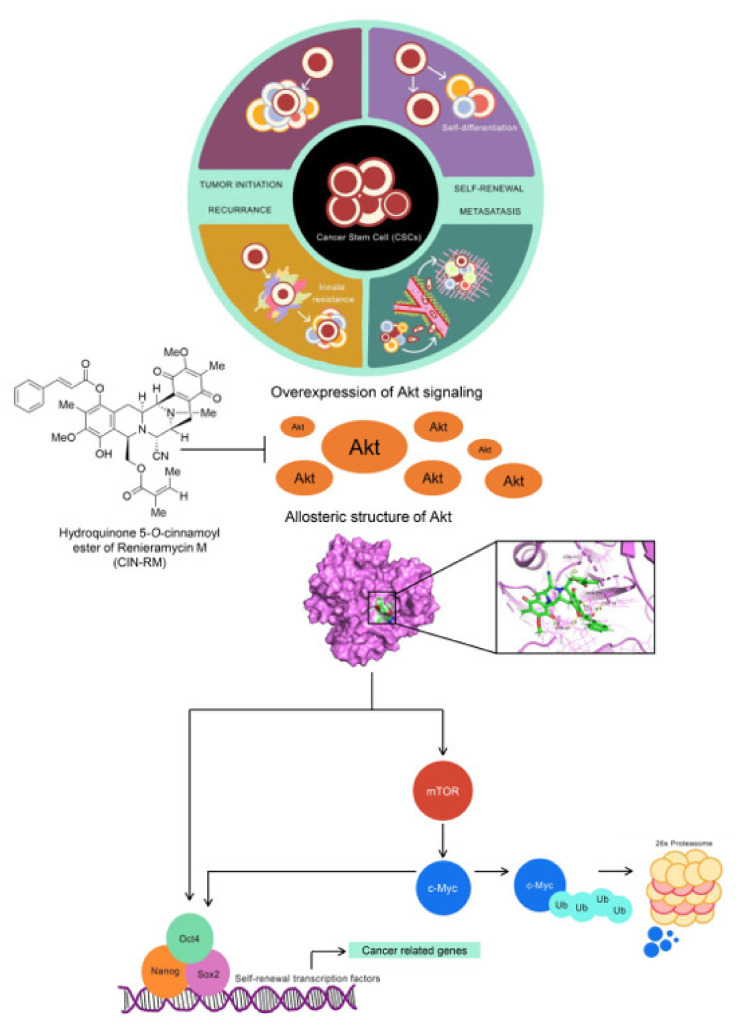
The proposed regulatory pathway is involved in the CSC suppression of CIN-RM. CSCs are the major cause of therapeutic failure due to their ability to self-renew and tumor initiation. They take part in cancer recurrence and metastasis. Akt signaling and related pathways are upregulated in CSCs leading to cancer aggressiveness. CIN-RM could directly interact and inhibit Akt function, resulting in the reduction of stem cell transcription factors. In addition, the inhibition of Akt triggers c-Myc proteasomal degradation. In the absence of upstream pluripotency factors Nanog, Oct4, and Sox2 and the protein co-factor c-Myc, the CSCs were depleted.

In conclusion, this study demonstrated that CIN-RM suppressed CSCs in H460 cells by inhibiting the AKT/c-Myc signaling pathway, resulting in the downregulation of the stem cell transcription factors, Nanog, Oct4, and Sox2 (Figure 8). Moreover, CIN-RM also suppresses the Akt regulating c-Myc pathway in other lung cancer cells. This study will be useful to further develop CIN-RM as an alternative treatment for CSCs in lung cancer.

## Figures and Tables

**Table 1:** The MM/GBSA (∆*G*_bind_) and its energy components (kcal/mol) of the CIN-RM/Akt complex.

CIN-RM/Akt (∆*G*_bind_)	
∆*E*_ele_	−18.4679 ± 4.0324
∆*E*_vdW_	−63.3923 ± 2.7583
∆*E*_MM_	−81.8602 ± 6.7907
∆*G*_solv,non-polar_	−8.3395 ± 0.2798
∆*G*_solv,polar_	41.3801 ± 4.1900
∆*G*_total_	−48.8170 ± 3.3050
−TDS	10.6352
∆*G*_bind_	−38.1818

## Data Availability

Data is contained within the article.

## Supplementary Materials

The following are available online at https://www.mdpi.com/article/10.3390/ph14111112/s1, Data S1: Spectroscopic data of CIN-RM structure.

## References

[1] Huang T., Song X., Xu D., Tiek D., Goenka A., Wu B., Sastry N., Hu B., Cheng S.-Y. (2020). Stem cell programs in cancer initiation, progression, and therapy resistance. Theranostics.

[2] Sowa T., Menju T., Sonobe M., Nakanishi T., Shikuma K., Imamura N., Motoyama H., Hijiya K., Aoyama A., Chen F. (2015). Association between epithelial-mesenchymal transition and cancer stemness and their effect on the prognosis of lung adenocarcinoma. Cancer Med..

[3] Yang L., Shi P., Zhao G., Xu J., Peng W., Zhang J., Zhang G., Wang X., Dong Z., Chen F. (2020). Targeting cancer stem cell pathways for cancer therapy. Signal Transduct. Target. Ther..

[4] Liu A., Yu X., Liu S. (2013). Pluripotency transcription factors and cancer stem cells: Small genes make a big difference. Chin. J. Cancer.

[5] Zhang H.F., Wu C., Alshareef A., Gupta N., Zhao Q., Xu X.E., Jiao J.W., Li E.M., Xu L.Y., Lai R. (2016). The PI3K/AKT/c-MYC axis promotes the acquisition of cancer stem-like features in esophageal squamous cell carcinoma. Stem Cells.

[6] Xia P., Xu X.-Y. (2015). PI3K/Akt/mTOR signaling pathway in cancer stem cells: From basic research to clinical application. Am. J. Cancer Res..

[7] Murphy M.J., Wilson A., Trumpp A. (2005). More than just proliferation: Myc function in stem cells. Trends Cell Biol..

[8] Elbadawy M., Usui T., Yamawaki H., Sasaki K. (2019). Emerging roles of C-Myc in cancer stem cell-related signaling and resistance to cancer chemotherapy: A potential therapeutic target against colorectal cancer. Int. J. Mol. Sci..

[9] Deb T.B., Coticchia C.M., Dickson R.B. (2004). Calmodulin-mediated activation of Akt regulates survival of c-Myc-overexpressing mouse mammary carcinoma cells. J. Biol. Chem..

[10] Chanvorachote P., Sriratanasak N., Nonpanya N. (2020). C-myc contributes to malignancy of lung Cancer: A potential anticancer drug target. Anticancer Res..

[11] Swords R.T., Schenk T., Stengel S., Gil V.S., Petrie K.R., Perez A., Ana R., Watts J.M., Vargas F., Elias R. (2015). Inhibition of the PI3K/AKT/mTOR pathway leads to down-regulation of c-Myc and overcomes resistance to ATRA in acute myeloid leukemia. Blood.

[12] Zhang X., Ai Z., Chen J., Yi J., Liu Z., Zhao H., Wei H. (2017). Glycometabolic adaptation mediates the insensitivity of drug-resistant K562/ADM leukaemia cells to adriamycin via the AKT-mTOR/c-Myc signalling pathway. Mol. Med. Rep..

[13] Chen H., Liu H., Qing G. (2018). Targeting oncogenic Myc as a strategy for cancer treatment. Signal Transduct. Target. Ther..

[14] Fagnocchi L., Zippo A. (2017). Multiple roles of MYC in integrating regulatory networks of pluripotent stem cells. Front. Cell Dev. Biol..

[15] Wang Z., Oron E., Nelson B., Razis S., Ivanova N. (2012). Distinct lineage specification roles for NANOG, OCT4, and SOX2 in human embryonic stem cells. Cell Stem Cell.

[16] Hu Y., Chen J., Hu G., Yu J., Zhu X., Lin Y., Chen S., Yuan J. (2015). Statistical research on the bioactivity of new marine natural products discovered during the 28 years from 1985 to 2012. Mar. Drugs.

[17] Khalifa S.A., Elias N., Farag M.A., Chen L., Saeed A., Hegazy M.-E.F., Moustafa M.S., El-Wahed A., Al-Mousawi S.M., Musharraf S.G. (2019). Marine natural products: A source of novel anticancer drugs. Mar. Drugs.

[18] Newman D.J., Cragg G.M. (2014). Marine-sourced anti-cancer and cancer pain control agents in clinical and late preclinical development. Mar. Drugs.

[19] Scott J.D., Williams R.M. (2002). Chemistry and biology of the tetrahydroisoquinoline antitumor antibiotics. Chem. Rev..

[20] Chamni S., Sirimangkalakitti N., Chanvorachote P., Suwanborirux K., Saito N. (2020). Chemistry of Renieramycins. Part 19: Semi-Syntheses of 22-O-Amino Ester and Hydroquinone 5-O-Amino Ester Derivatives of Renieramycin M and Their Cytotoxicity against Non-Small-Cell Lung Cancer Cell Lines. Mar. Drugs.

[21] Sirimangkalakitti N., Chamni S., Suwanborirux K., Chanvorachote P. (2017). Renieramycin m attenuates cancer stem cell-like phenotypes in h460 lung cancer cells. Anticancer Res..

[22] Maiuthed A., Pinkhien T., Chamni S., Suwanborirux K., Saito N., Petpiroon N., Chanvorachote P. (2017). Apoptosis-inducing effect of hydroquinone 5-o-cinnamoyl ester analog of renieramycin m on non-small cell lung cancer cells. Anticancer Res..

[23] Chamni S., Sirimangkalakitti N., Chanvorachote P., Saito N., Suwanborirux K. (2017). Chemistry of renieramycins. 17. A new generation of renieramycins: Hydroquinone 5-O-monoester analogues of renieramycin M as potential cytotoxic agents against non-small-cell lung cancer cells. J. Nat. Prod..

[24] Mori S., Chang J.T., Andrechek E.R., Matsumura N., Baba T., Yao G., Kim J.W., Gatza M., Murphy S., Nevins J.R. (2009). Anchorage-independent cell growth signature identifies tumors with metastatic potential. Oncogene.

[25] Farrell A.S., Sears R.C. (2014). MYC degradation. Cold Spring Harb. Perspect. Med..

[26] Mossahebi-Mohammadi M., Quan M., Zhang J.-S., Li X. (2020). FGF signaling pathway: A key regulator of stem cell pluripotency. Front. Cell Dev. Biol..

[27] Yu Z., Pestell T.G., Lisanti M.P., Pestell R.G. (2012). Cancer stem cells. Int. J. Biochem. Cell Biol..

[28] Reya T., Morrison S.J., Clarke M.F., Weissman I.L. (2001). Stem cells, cancer, and cancer stem cells. Nature.

[29] Würth R., Barbieri F., Florio T. (2014). New molecules and old drugs as emerging approaches to selectively target human glioblastoma cancer stem cells. BioMed Res. Int..

[30] Radke J., Bortolussi G., Pagenstecher A. (2013). Akt and c-Myc induce stem-cell markers in mature primary p53^−/−^ astrocytes and render these cells gliomagenic in the brain of immunocompetent mice. PLoS ONE.

[31] Quan Y., Wang N., Chen Q., Xu J., Cheng W., Di M., Xia W., Gao W.-Q. (2015). SIRT3 inhibits prostate cancer by destabilizing oncoprotein c-MYC through regulation of the PI3K/Akt pathway. Oncotarget.

[32] Chantarawong W., Chamni S., Suwanborirux K., Saito N., Chanvorachote P. (2019). 5-O-Acetyl-Renieramycin T from Blue Sponge Xestospongia sp. Induces Lung Cancer Stem Cell Apoptosis. Mar. Drugs.

[33] Ottinger S., Klöppel A., Rausch V., Liu L., Kallifatidis G., Gross W., Gebhard M.M., Brümmer F., Herr I. (2012). Targeting of pancreatic and prostate cancer stem cell characteristics by Crambe crambe marine sponge extract. Int. J. Cancer.

[34] Suresh R., Ali S., Ahmad A., Philip P.A., Sarkar F.H. (2016). The role of cancer stem cells in recurrent and drug-resistant lung cancer. Lung Cancer and Personalized Medicine: Novel Therapies and Clinical Management.

[35] Sullivan J.P., Spinola M., Dodge M., Raso M.G., Behrens C., Gao B., Schuster K., Shao C., Larsen J.E., Sullivan L.A. (2010). Aldehyde dehydrogenase activity selects for lung adenocarcinoma stem cells dependent on notch signaling. Cancer Res..

[36] Huang C.-P., Tsai M.-F., Chang T.-H., Tang W.-C., Chen S.-Y., Lai H.-H., Lin T.-Y., Yang J.C.-H., Yang P.-C., Shih J.-Y. (2013). ALDH-positive lung cancer stem cells confer resistance to epidermal growth factor receptor tyrosine kinase inhibitors. Cancer Lett..

[37] Schaefer T., Steiner R., Lengerke C. (2020). SOX2 and p53 expression control converges in PI3K/AKT signaling with versatile implications for stemness and cancer. Int. J. Mol. Sci..

[38] Srinual S., Chanvorachote P., Pongrakhananon V. (2017). Suppression of cancer stem-like phenotypes in NCI-H460 lung cancer cells by vanillin through an Akt-dependent pathway. Int. J. Oncol..

[39] Su T., Dan S., Wang Y. (2014). Akt–Oct4 regulatory circuit in pluripotent stem cells. Chin. Sci. Bull..

[40] Zayed H., Petersen I. (2018). Stem cell transcription factor SOX2 in synovial sarcoma and other soft tissue tumors. Pathol.-Res. Pract..

[41] Lin Y., Yang Y., Li W., Chen Q., Li J., Pan X., Zhou L., Liu C., Chen C., He J. (2012). Reciprocal regulation of Akt and Oct4 promotes the self-renewal and survival of embryonal carcinoma cells. Mol. Cell.

[42] Takahashi K., Yamanaka S. (2006). Induction of pluripotent stem cells from mouse embryonic and adult fibroblast cultures by defined factors. Cell.

[43] Gustafson W., Weiss W. (2010). Myc proteins as therapeutic targets. Oncogene.

[44] Liu G., Shi A., Wang N., Li M., He X., Yin C., Tu Q., Shen X., Tao Y., Wang Q. (2020). Polyphenolic Proanthocyanidin-B2 suppresses proliferation of liver cancer cells and hepatocellular carcinogenesis through directly binding and inhibiting AKT activity. Redox Biol..

[45] Elghazi L., Balcazar N., Bernal-Mizrachi E. (2006). Emerging role of protein kinase B/Akt signaling in pancreatic β-cell mass and function. Int. J. Biochem. Cell Biol..

[46] Nitulescu G.M., Margina D., Juzenas P., Peng Q., Olaru O.T., Saloustros E., Fenga C., Spandidos D.A., Libra M., Tsatsakis A.M. (2016). Akt inhibitors in cancer treatment: The long journey from drug discovery to clinical use. Int. J. Oncol..

[47] Suwanborirux K., Amnuoypol S., Plubrukarn A., Pummangura S., Kubo A., Tanaka C., Saito N. (2003). Chemistry of renieramycins. Part 3. Isolation and structure of stabilized renieramycin type derivatives possessing antitumor activity from Thai sponge Xestospongia species, pretreated with potassium cyanide. J. Nat. Prod..

[48] Kaur K., Kaur P., Mittal A., Nayak S.K., Khatik G.L. (2017). Design and molecular docking studies of novel antimicrobial peptides using autodock molecular docking software. Asian J. Pharm. Clin. Res..

[49] O’Boyle N.M., Banck M., James C.A., Morley C., Vandermeersch T., Hutchison G.R. (2011). Open Babel: An open chemical toolbox. J. Cheminformatics.

[50] Wu W.-I., Voegtli W.C., Sturgis H.L., Dizon F.P., Vigers G.P., Brandhuber B.J. (2010). Crystal structure of human AKT1 with an allosteric inhibitor reveals a new mode of kinase inhibition. PLoS ONE.

[51] Morris G.M., Huey R., Lindstrom W., Sanner M.F., Belew R.K., Goodsell D.S., Olson A.J. (2009). AutoDock4 and AutoDockTools4: Automated docking with selective receptor flexibility. J. Comput. Chem..

[52] Trott O., Olson A.J. (2010). AutoDock Vina: Improving the speed and accuracy of docking with a new scoring function, efficient optimization, and multithreading. J. Comput. Chem..

[53] Guex N. (1996). Swiss-PdbViewer: A fast and easy-to-use PDB viewer for Macintosh and PC. Protein Data Bank Q. Newsl..

[54] Hanwell M.D., Curtis D.E., Lonie D.C., Vandermeersch T., Zurek E., Hutchison G.R. (2012). Avogadro: An advanced semantic chemical editor, visualization, and analysis platform. J. Cheminformatics.

[55] Da Silva A.W.S., Vranken W.F. (2012). ACPYPE-Antechamber python parser interface. BMC Res. Notes.

[56] Wang J., Wolf R.M., Caldwell J.W., Kollman P.A., Case D.A. (2004). Development and testing of a general amber force field. J. Comput. Chem..

[57] Maier J.A., Martinez C., Kasavajhala K., Wickstrom L., Hauser K.E., Simmerling C. (2015). ff14SB: Improving the accuracy of protein side chain and backbone parameters from ff99SB. J. Chem. Theory Comput..

[58] Jorgensen W.L., Chandrasekhar J., Madura J.D., Impey R.W., Klein M.L. (1983). Comparison of simple potential functions for simulating liquid water. J. Chem. Phys..

[59] Bussi G., Donadio D., Parrinello M. (2007). Canonical sampling through velocity rescaling. J. Chem. Phys..

[60] Essmann U., Perera L., Berkowitz M.L., Darden T., Lee H., Pedersen L.G. (1995). A smooth particle mesh Ewald method. J. Chem. Phys..

[61] Hess B., Bekker H., Berendsen H.J., Fraaije J.G. (1997). LINCS: A linear constraint solver for molecular simulations. J. Comput. Chem..

[62] Abraham M.J., Murtola T., Schulz R., Páll S., Smith J.C., Hess B., Lindahl E. (2015). GROMACS: High performance molecular simulations through multi-level parallelism from laptops to supercomputers. SoftwareX.

[63] Genheden S., Ryde U. (2015). The MM/PBSA and MM/GBSA methods to estimate ligand-binding affinities. Expert Opin. Drug Discov..

[64] Miller B.R., McGee T.D., Swails J.M., Homeyer N., Gohlke H., Roitberg A.E. (2012). MMPBSA.py: An efficient program for end-state free energy calculations. J. Chem. Theory Comput..

[65] Pawar S.S., Rohane S.H. (2021). Review on Discovery Studio: An important Tool for Molecular Docking. Asian J. Res. Chem..

