# Supplementary material for: Hydroquinone 5-O-Cinnamoyl Ester of Renieramycin M Suppresses Lung Cancer Stem Cells by Targeting Akt and Destabilizes c-Myc
Source: Pharmaceuticals (Basel). 2021 Oct 30;14(11):1112. doi: 10.3390/ph14111112 (PMC8621304; doi:10.3390/ph14111112)
Supplement: Supplementary file 1 [file pharmaceuticals-14-01112-s001.zip › pharmaceuticals-1411067-supplementary.pdf]

Supplement material

Data S1. Spectroscopic data of CIN-RM structure.

^{1}H-NMR ($CDCl_3$, 400 MHz) δ in ppm: 7.81 (1H, d, *J* = 16.0 Hz, CH=CH_β), 7.62 (2H, m, H-3′and H-5′), 7.46 (3H, m, H-2′, H-6′, and H-4′), 6.67 (1H, d, *J* = 16.0 Hz, CH_α=CH), 6.01 (1H, qq, *J* = 7.4, 1.2 Hz, H-26), 5.79 (1H, s, OH-8), 4.54 (1H, d, *J* = 11.2 Hz, H-22b), 4.34 (1H, br t, *J* = 4.0 Hz, H-1), 4.13 (1H, d, *J* = 2.0 Hz, H-21), 4.07 (1H, dd, *J* = 11.2, 4.0 Hz, H-22a), 3.99 (1H, br d, *J* = 3.6 Hz, H-11), 3.85 (3H, s, 17-OCH_3), 3.80 (3H, s, 7-OCH_3), 3.36 (1H, dt, *J* = 7.2, 2.0 Hz, H-13), 3.22 (1H, d, *J* = 12.0 Hz, H-3), 2.74 (1H, dd, *J* = 20.8, 7.2 Hz, H-14*α*), 2.60 (1H, dd, *J* = 14.0, 2.0 Hz, H-4*α*), 2.29 (1H, d, *J* = 20.8 Hz, H-14*β*), 2.25 (3H, s, 12-NCH_3), 2.12 (3H, s, 6-CH_3), 1.89 (3H, dq, *J* = 7.2, 1.4 Hz, H_3-27), 1.90 (3H, s, 16-CH_3), 1.72 (3H, dq, *J* = 1.4, 1.2 Hz, H_3-28), 1.66 (1H, overlapped, H-4*β*); ^{13}C-NMR ($CDCl_3$, 100 MHz) δ in ppm: 186.0 (C, C-15), 182.6 (C, C-18), 167.1 (C, C-24), 164.7 (C, 5-O*C*=O), 155.5 (C, C-17), 146.8 (CH, CH=CH_β), 143.8 (C, C-7), 143.1 (C, C-8), 141.5 (C, C-20), 140.0 (CH, C-26), 139.1 (C, C-5), 135.7 (C, C-19), 134.1 (C, C-1′), 131.0 (CH, C-4′), 129.1 (CH, C-2′ and C-6′), 128.8 (C, C-16), 128.4 (CH, C-3′ and C-5′), 126.8 (C, C-25), 124.5 (C, C-10), 122.6 (C, C-6), 117.6 (C, 21-CN), 117.2 (C, C-9), 116.4 (CH, CH_α=CH), 64.5 (CH_2, C-22), 61.2 (CH_3, 7-OCH_3), 60.6 (CH_3, 17-OCH_3), 59.5 (CH, C-21), 56.5 (CH, C-1), 55.3 (CH, C-3), 54.9 (CH, C-13), 54.6 (CH, C-11), 41.5 (CH_3, 12-NCH_3), 27.8 (CH_2, C-4), 21.1 (CH_2, C-14), 20.6 (CH_3, C-28), 15.9 (CH_3, C-27), 10.1 (CH_3, 6-CH_3), 8.6 (CH_3, 16-CH_3).
